# Accumulating the hydride state in the catalytic cycle of [FeFe]-hydrogenases

**DOI:** 10.1038/ncomms16115

**Published:** 2017-07-19

**Authors:** Martin Winkler, Moritz Senger, Jifu Duan, Julian Esselborn, Florian Wittkamp, Eckhard Hofmann, Ulf-Peter Apfel, Sven Timo Stripp, Thomas Happe

**Affiliations:** 1 AG Photobiotechnologie, Lehrstuhl für Biochemie der Pflanzen, Fakultät für Biologie und Biotechnologie, Ruhr-Universität Bochum, Universitätsstraße 150, 44801 Bochum, Germany; 2 Experimental Molecular Biophysics, Department of Physics, Freie Universität Berlin, Arnimallee 14, 14195 Berlin, Germany; 3 Fakultät für Chemie und Biochemie, Lehrstuhl für Anorganische Chemie I/Bioanorganische Chemie, Ruhr-Universität Bochum, Universitätsstraße 150, 44801 Bochum, Germany; 4 AG Proteinkristallographie, Lehrstuhl für Biophysik, Fakultät für Biologie und Biotechnologie, Ruhr-Universität Bochum, Universitätsstraße 150, 44801 Bochum, Germany

## Abstract

H_2_ turnover at the [FeFe]-hydrogenase cofactor (H-cluster) is assumed to follow a reversible heterolytic mechanism, first yielding a proton and a hydrido-species which again is double-oxidized to release another proton. Three of the four presumed catalytic intermediates (H_ox_, H_red_/H_red_ and H_sred_) were characterized, using various spectroscopic techniques. However, in catalytically active enzyme, the state containing the hydrido-species, which is eponymous for the proposed heterolytic mechanism, has yet only been speculated about. We use different strategies to trap and spectroscopically characterize this transient hydride state (H_hyd_) for three wild-type [FeFe]-hydrogenases. Applying a novel set-up for real-time attenuated total-reflection Fourier-transform infrared spectroscopy, we monitor compositional changes in the state-specific infrared signatures of [FeFe]-hydrogenases, varying buffer pH and gas composition. We selectively enrich the equilibrium concentration of H_hyd_, applying Le Chatelier’s principle by simultaneously increasing substrate and product concentrations (H_2_/H^+^). Site-directed manipulation, targeting either the proton-transfer pathway or the adt ligand, significantly enhances H_hyd_ accumulation independent of pH.

## Abstract (web summary)

Of the four intermediates in the catalytic cycle of [FeFe]-hydrogenases, the hydride state still has eluded characterization. Here, the authors shift the reaction equilibrium for three hydrogenases and enrich the hydride-bound species, characterizing them using a real-time IR spectroscopic technique.

Hydrogen (H_2_) turnover in [FeFe]-hydrogenases is proposed to occur at the distal iron site (Fe_d_) of the [2Fe2S] moiety (2Fe_H_), which is part of the H-cluster^1^. The distal iron ion exchanges protons with a conserved proton-transfer pathway (PTP)^2^,^3^ via an azadithiolate ligand (adt) that bridges both iron sites (Fig. 1)^4^,^5^. Different redox states of the H-cluster can be distinguished by the unique infrared vibrational signatures of the two CN^−^ and three CO ligands at the 2Fe_H_ moiety. To tailor [FeFe]-hydrogenases for specific applications or translate their catalytic principle into productive and durable synthetic mimics, detailed knowledge of the succession of turnover steps occurring at the H-cluster is required. The binuclear metal cofactors of [FeFe]- and [NiFe]-hydrogenases have been suggested to oxidize H_2_ by enhancing the acidity of H_2_ in the presence of a base, thus facilitating its heterolytic cleavage into a proton (H^+^) and hydride (H^−^) as a first intermediate step (Fig. 1)^6^,^7^,^8^,^9^.

For [NiFe]-hydrogenases, electron paramagnetic resonance spectroscopy showed that a bridging metal hydride was present in the paramagnetic Ni-C state^6^,^7^, while an ultra-high 0.89 Å resolution X-ray crystallography structure enabled the assignment of both, the proton and bridging hydride, in the first state following H_2_-binding (Ni-R)^8^. For algal-type [FeFe]-hydrogenases, a comparative density functional theory (DFT)/X-ray absorption spectroscopy analysis indicated a bridging hydride (μ-H^−^) in the double-reduced H_sred_ state^10^, albeit the importance of a μ-H^−^ state for fast catalytic turnover has been questioned. In fact, the terminal hydride (t-H^−^) is thermodynamically less stable and more reactive compared to μ-H^−^ and would match the high turnover kinetics of [FeFe]-hydrogenases far better^11^,^12^,^13^,^14^. According to the current working model for the catalytic mechanism of [FeFe]-hydrogenases^1^, binding of H_2_ to the oxidized active ready state (H_ox_) results in the heterolytic cleavage of H_2_, with H_hyd_ as the first intermediate. On deprotonation, H_hyd_ is converted into the rather stable H_sred_ state before two successive oxidation steps recycle H_ox_. A direct conversion of H_hyd_ into H_red_ has been proposed as well^15^. However, a terminal hydride has not been assigned to any of the known redox states^10^, nor has another catalytic wild-type state with the postulated t-H^−^ been reported. This implies that the kinetically relevant hydride state is a transient one and difficult to trap under steady-state conditions. Owing to the rules governing steady-state kinetics, which favour thermodynamically stable intermediates in catalysis, the direct characterization of transient states relies on the utilization of time-resolved approaches with stopped-flow or single-turnover set-ups^16^.

We take a different approach, exploiting the properties of steady-state kinetics and using Le Chatelier’s principle to selectively enrich the transient t-H^−^ state in three different [FeFe]-hydrogenases: HydA1; DdH; and CpI, which cover the complete range of increasingly complex structured monomeric hydrogenase subtypes (M1–M3)^17^,^18^,^19^,^20^. In our experimental set-up, attenuated total reflection (ATR) Fourier-transform infrared (FTIR) spectroscopy is used to follow the population of different catalytic states. Compositional changes are monitored in the state-specific infrared signatures as a function of buffer/gas composition.

## Results

### Accumulation of H_hyd_ in wild-type [FeFe]-hydrogenases

Figure 2a depicts FTIR difference spectra of HydA1 film at pH 8 recorded while changing the atmosphere from N_2_ to H_2_ (for details about the experimental setup see Supplementary Fig. 1). Negative bands mainly comprised the frequency pattern of H_ox_ (1,964/1,940/1,802 cm^−1^). Positive bands included marker frequencies 1,915/1,891 cm^−1^ (H_red_) and 1,953/1,918/1,882 cm^−1^ (H_sred_), corresponding to earlier measurements with H_2_-purged HydA1 carried out in transmission FTIR systems^21^. On steady-state supply of substrate (that is, H_2_), the enzyme accumulates the apparently rather stable reduced intermediates H_red_ and H_sred_. However, bands at 1,978/1,960/1,860 cm^−1^ could be identified as minor contributions, indicating the presence of another state, which resembles the band pattern of H_trans_ in DdH (H_trans_-like state)^22^ and was previously observed as a weak fraction of a complex band pattern in a sodium dithionite (NaDT)-treated HydA1 sample^23^. According to our working model, deprotonation should lead from the presupposed H_hyd_ to the reduced states (Fig. 2b), and a plausible way to selectively enrich H_hyd_ would be a simultaneous increase in substrate (H_2_) and product (H^+^) pressure (Fig. 2d). We therefore titrated HydA1 to pH 4 before H_2_ exposure, enhancing the proton pressure by four orders of magnitude (Fig. 2c). The presence of a band at 1,891 cm^−1^ in the resulting difference spectrum hinted at a minor contribution of H_red_, while no H_sred_ could be detected. Instead, the H_trans_-like state represented the main fraction of our protein sample. No loss of cofactor was observed in the time frame of hours (Supplementary Fig. 2a,b), and the back-titration of HydA1 sample buffer from pH 4 to pH 8 clearly demonstrates a pH-dependent reversibility of the indicated state transitions (Supplementary Fig. 2b,c). The same applies for H_2_-dependency at pH 4 (Supplementary Fig. 2d). This bi-directionality is not compatible with the supposed H_trans_-like nature of the novel pattern^22^. Therefore, we will refer to this redox state in the following as H_hyd_. Similar results were obtained when repeating this experiment with samples of native DdH and CpI as representatives of the other two major [FeFe]-hydrogenase subtypes, implying this state and behaviour to be general features of the [FeFe]-hydrogenase family (Supplementary Fig. 3).

### The influence of proton-transfer efficiency

As the H_2_/H^+^ balance appears to affect the electronic configuration of the H-cluster, alternative approaches were designed to enrich H_hyd_. HydA1 sample was once more purged with H_2_ aerosol to first enrich the reduced states. When humidity was removed from the H_2_ gas stream, sample films reacted by rapid dehydration. As predicted, this as well leads to a loss of H_red_/H_sred_ in favour of the 1,978/1,960/1,860 cm^−1^ pattern (Supplementary Fig. 4a) indicating that the loss of bulk water has a similar effect to lowering the buffer pH, likely due to subsiding proton-transfer efficiency^24^ (Supplementary Fig. 4).

**Figure 1:**
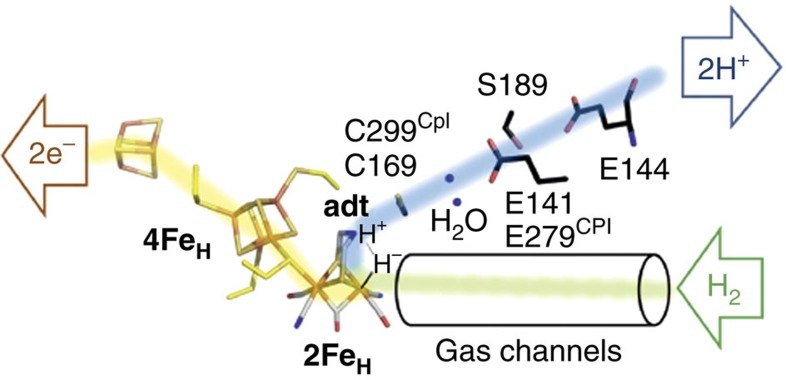
Substrate/product transfer and heterolytic H_2_ splitting in [FeFe]-hydrogenases. The electron-transfer path is shown in yellow, the PTP is depicted in blue. H_2_ is released from, or reaches the 2Fe_H_ cluster through hydrophobic gas channels (green). At the catalytically pivotal 2Fe_H_ site, the presumptive first step in the catalysis of H_2_ oxidation (H_2_ heterolysis) is depicted, resulting in the unequal intermediates H^+^, binding at the adt-bridge (Lewis base) and H^−^, binding at Fe_d_ (Lewis acid).

To verify that an interference with the PTP enhances the population of the H_hyd_ state under H_2_ exposure, we blocked the PTP in HydA1 and CpI via site-directed mutagenesis at two strictly conserved positions for each protein. Position 1 (C169 of HydA1/C299 of CpI; Fig. 1), which was shown in earlier experimental data to be involved in catalytic proton transfer^3^,^5^, marks the most proximal position of the PTP to the 2Fe_H_ sub-cluster, while position 2 (E141 of HydA1/E279 of CpI; Fig. 1) is situated more distant from the H-cluster. An exchange to alanine almost completely abolished H_2_-release activity for all variants (Supplementary Fig. 6). To probe the structural integrity and unequivocally connect the monitored effects to an interrupted PTP, we solved the crystal structure of CpI E279A (Fig. 3). The X-ray structure illustrates a fully intact H-cluster and overall identical structural features compared to wild-type CpI (refs 19, 25), with the exception of the clearly diminished electron density at position 279, which is in full accordance with an exchange from glutamate to alanine. As a consequence of this non-conservative exchange, the path of polar side chains is interrupted between S319 and the harnessed H_2_O molecule. Figure 4a,b shows the reaction of enzyme variants C169A of HydA1 and E279A of CpI towards H_2_ (black graph) at pH 8. Similar to the effect of acidification or sample drying, the oxidized state is lost in favour of bands at 1,978/1,962/1,863 cm^−1^ or 1,985/1,970/1,858 cm^−1^, respectively.

We further exploited the effect of H_hyd_ accumulation as a consequence of PTP obstruction to elucidate the proposed role of the azadithiolate ligand in the heterolytic cleavage of H_2_. The earlier-described procedure of *in vitro* maturation allows for the replacement of the native 2Fe_H_ site with derivatives such as odt-2Fe_H_, which contains an oxodithiolate group ((SCH_2_)_2_O,odt) instead of the native azadithiolate ((SCH_2_)_2_NH, adt)^4^,^25^,^26^. After *in vitro* maturation with odt-2Fe_H_ in presence of H_2_, HydA1^odt^ exhibits an infrared band pattern that strongly resembles H_hyd_ (refs 23, 26). The oxidized state of HydA1^odt^ was obtained via extensive purging with humidified N_2_. Subsequent exposure to H_2_ induced a decrease in the H_ox_ pattern in favour of bands 1,980, 1,962 and 1868, cm^−1^ (Fig. 4c, black graph), however at a significantly lower rate compared to the corresponding experiment with wild-type HydA1.

### A terminal hydride in wild-type [FeFe]-hydrogenases

To confirm the presence of a terminal hydride (t-H^−^) in H_hyd_ for wild-type hydrogenase and all examined variants, we probed the effect of an H/D isotope exchange on the FTIR band position of the bridging CO ligand, μCO (see Fig. 4, red graph, and Supplementary Figs 7 and 8 including Supplementary Note 1). Terminal hydride species on metal carbonyl complexes are vibrationally coupled with the ligand positioned in *trans* due to resonant frequencies^27^. Previously published DFT calculations on the H-cluster model of HydA1 variant C169S predict a selective ‘redshift’ of 6 cm^−1^ for the μCO frequency as a consequence of substituting a hydride for a deuteride species located *trans* to μCO (ref. 23). When switching the purging gas from H_2_ to D_2_, CpI E279A (Fig. 4a) and HydA1 C169A (Fig. 4b) showed a selective shift to lower energies by 5–6 cm^−1^ of the band at 1,860 cm^−1^, which corresponds to the stretching vibration of μCO. A similar ‘redshift’ was observed for HydA1^odt^ when the oxidized enzyme was extensively treated with D_2_ (Fig. 4c and Supplementary Fig. 7b). In Supplementary Fig. 8 we show spectra that document the H/D effect on wild-type HydA1 in presence of pH 4 and H_2_/D_2_. This confirms the corresponding behaviour of the H_hyd_ state for all protein variants examined here.

## Discussion

Le Chatelier’s principle was applied to enrich the highly transient H-cluster intermediate H_hyd_, which according to the current working model of catalytic H_2_ turnover is predicted to carry a terminal hydride species. By simultaneously enhancing substrate and product pressure, we accumulated H_hyd_ for three different wild-type [FeFe]-hydrogenases. Interestingly, a nearly identical FTIR band pattern was observed in our earlier spectroscopic analysis of the largely inactive HydA1 PTP variant C169S (ref. 28). Using DFT calculations, Mulder *et al*.^15^,^23^ assigned this band pattern and redox species to a model of C169S that carried a terminal hydride at Fe_d_ (Supplementary Note 1 and Supplementary Fig. 5).

Accumulation of H_hyd_ was also observed on decreasing the humidity level of the protein sample. Recently, we demonstrated that freeze-drying renders [FeFe]-hydrogenase samples insensitive to O_2_ exposure^24^. Although X-ray absorption spectroscopy data showed the formation of a stable O_2_ adduct at the 2Fe_H_ site, H-cluster degradation was not initiated, as opposed to hydrated enzyme in presence of O_2_ (ref. 29). DFT calculations suggested a protonation step mandatory for generating destructive reactive oxygen species^30^. Therefore, a lack of H^+^-transfer activity in the freeze-dried state appeared to be a plausible explanation for this effect and dehydration should have a similar effect as lowering the pH. It can be speculated that the loss of bulk water, being the acceptor pool for exported protons, leads to an accumulation of protons in the PTP from the inside. Accordingly, low pH and dehydration both provide a counter pressure that prevents the H_2_-activated state from being deprotonated and proceeding to the reduced states (Supplementary Fig. 4b,c).

**Figure 2:**
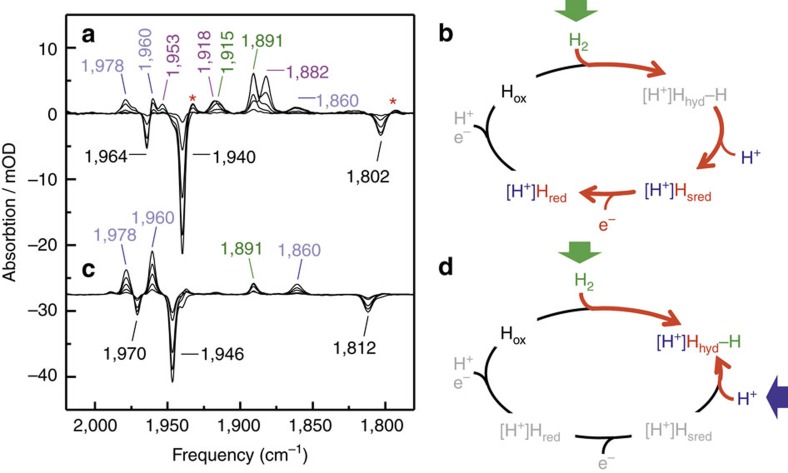
Population of H_hyd_ in HydA1 by simultaneously increasing H_2_ and H^+^ pressure. (**a**) ATR FTIR difference spectrum (N_2_/pH8→H_2_/pH8), depicting the accumulation of the reduced states (H_red_/H_red_ and H_sred_) in the catalytic cycle of HydA1 under H_2_ at pH 8. (**c**) ATR FTIR difference spectrum (N_2_/pH8→H_2_/pH4) depicting the accumulation of H_hyd_ in the catalytic cycle of HydA1 under H_2_ at pH 4. Catalytic cycles (**b**,**d**) beside the corresponding spectra illustrate the shift in steady-state equilibrium depending on applied substrate and product pressures in the working model of the catalytic cycle, which comprises the already-characterized states H_ox_, H_red_/H_red_ and H_sred_, and the missing H_2_-activated state (H_hyd_). Peak labels for H_ox_ are presented in black, for H_hyd_ in blue, for H_red_ in green and for H_sred_ in magenta (red asterisks: H_red_). For the complete state-specific CN^−^-/CO-vibrational spectra of HydA1 observed during sample analysis, see Supplementary Table 1. At pH 4, the vibrational signals of H_ox_ were slightly shifted to higher frequencies (for more information see Supplementary Table 1, Hox-blue).

**Figure 3:**
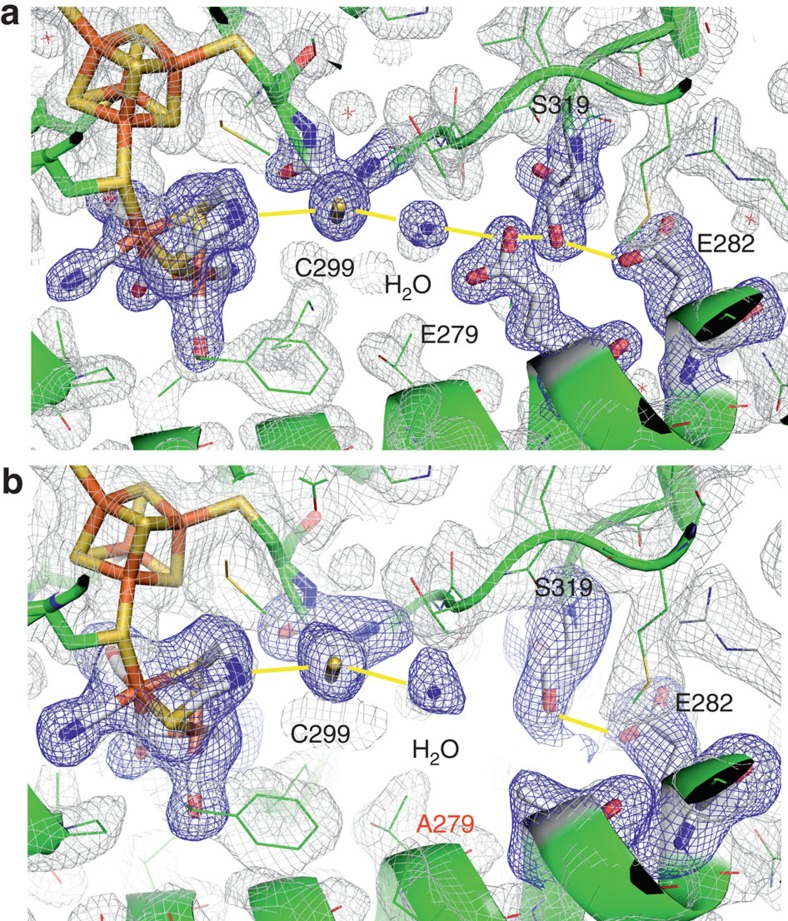
Crystal structure of the PTP in wild-type CpI and PTP-defective CpI variant E279A. Stick models and simulated annealing omit |Fo–Fc| electron density maps (blue) of the 2Fe_H_ cluster, and the amino-acid residues and the H_2_O molecules involved in proton transfer embedded in the 2Fo–Fc electron density maps (grey) of the X-ray structures of CpI wild-type^25^ (**a**) and variant E279A (**b**). The presumed H^+^-transfer pathway is indicated in yellow. Crystal structure data for wild-type CpI correspond to pdb database entry 4XDC^25^. Details of the crystallographic data of E279A are summarized in Supplementary Table 2. For stereo view presentations see Supplementary Fig. 9.

The N_2_/H_2_ difference spectra of site-directed exchange variants of HydA1 and CpI targeting positions C169/C299 and E141/E279 clearly demonstrate how the disrupted proton transport stabilizes the H_hyd_ state regardless of buffer pH. This highlights that the transient character of H_hyd_ essentially depends on a functional catalytic proton transfer, just as predicted from the working model (Figs 1 and 2). In return, the analogous behaviour of cysteine and glutamate variants provides experimental proof for the involvement of the median glutamic acid residue (E141^HydA1^/E279^CpI^) in the proton-transfer mechanism of [FeFe]-hydrogenases. In future mutagenesis studies on the PTP of [FeFe]-hydrogenases, accumulation of H_hyd_ can be regarded as an experimental indicator for the involvement of peptide positions in the catalytic H^+^ transfer.

**Figure 4:**
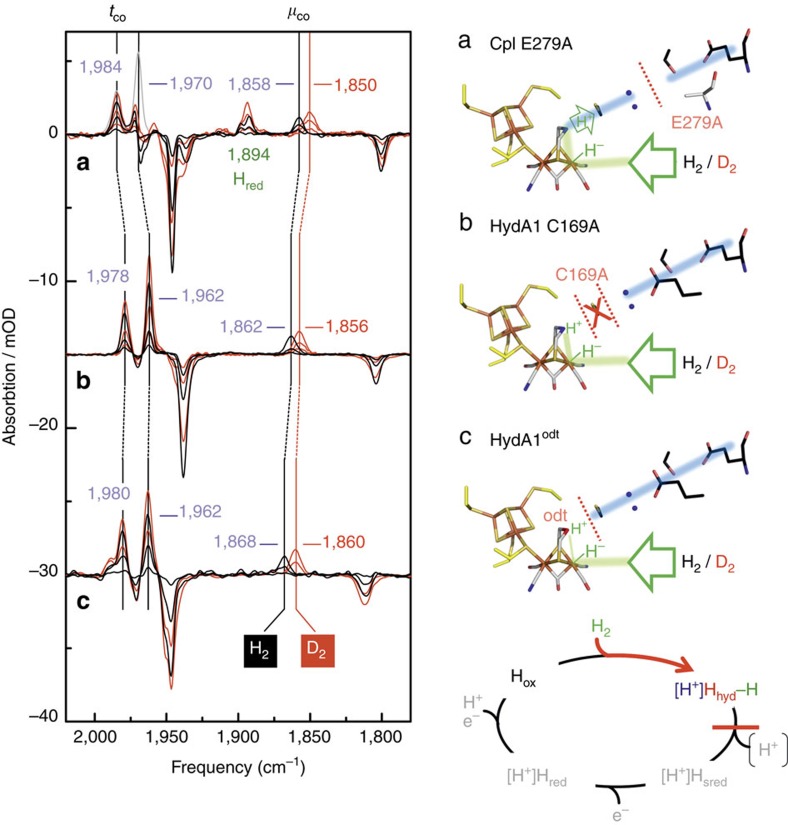
Manipulating H^+^ transfer traps the H_hyd_ state under H_2_ or D_2_ regardless of buffer pH. Left: ATR FTIR difference spectra (N_2_/pH8→H_2_ or D_2_/pH8) depicting the accumulation of H_hyd_ for variants of the PTP E279A (CpI) (**a**) and C169A (HydA1) (**b**), as well as HydA1^odt^ (**c**) under H_2_ or D_2_ at pH 8. The grey spectrum in **a** depicts the absolute absorption bands of H_hyd_. Negative bands are assigned to H_ox_ (Supplementary Table 1). Right: location of the corresponding PTP manipulation illustrated in a crystal structure model of CpI (pdb ID: 4XDC). Accumulation of H_hyd_ under D_2_ (red spectra) does not affect the tCO-frequencies but induces a selective redshift for the μCO signal of 6–8 cm^−1^ in all analysed variants. For the complete state-specific CN^−^/CO-vibrational spectra observed during sample analysis, see Supplementary Table 1.

The relevance of the bridgehead position of the 2Fe_H_ subcluster for catalytic proton transfer was clearly demonstrated by the fact that cofactor variant HydA1^odt^ could be trapped in H_hyd_ under H_2_ independent of the pH, thus confirming the results of earlier studies, which pointed out the essential role of the native adt ligand as a proton relay to the substrate-binding site at Fe_d_. The fact that H_2_ exposure of HydA1^odt^ results in H_hyd_ accumulation suggests that the ether bridgehead of odt is capable to serve as a Lewis base and supports heterolytic H_2_ cleavage. This reflects earlier investigations of Barton *et al*.,^31^ which demonstrated in a comparative study, including different diiron complexes that the weakly basic ether group of odt can assist in proton relay during H_2_ evolution^31^. However, in contrast to C169A and E279A, the H_hyd_ spectrum of HydA1^odt^ could only be regained very slowly in presence of H_2_. While all examined enzyme variants exhibited an impaired proton transfer, for C169A and E279A the defect was restricted to the H^+^ transfer across the protein shell (Fig. 4). In case of HydA1^odt^, the impairment already affected proton abstraction during H_2_ heterolysis. This finding experimentally verifies the hypothesis that the pending amine group in the native adt ligand significantly enhances the rate of heterolytic H_2_ cleavage^31^. The selective shift of the μCO band observed in H_hyd_ on H/D exchange suggests the presence of a terminal hydride ligand for all PTP variants and catalytically competent wild-type enzymes examined here.

In addition, two recently published studies independently demonstrated a terminal hydride for inactive HydA1 variants assigned to the presumptive H_hyd_ state. Reijerse *et al*.^32^ reported the direct detection of Fe_d_-H bending vibrations via nuclear resonance vibrational spectroscopy of a terminal hydride species in HydA1^odt^ after isolation under H_2_. This is in good agreement with our real-time analysis of HydA1^odt^. Furthermore, Mulder *et al*.^15^ applied electron paramagnetic resonance and Mössbauer spectroscopy to provide experimental support for the presence of a metal hydride species in HydA1 variant C169S, which largely adopted H_hyd_ on supplementation with NaDT. Well in agreement with electrochemistry studies on HydA1 under turnover conditions^14^, the authors determined a H_hyd_ transition potential close to the H_2_/H^+^ redox couple. This argues in favour of the relevance of H_hyd_ in the catalytic cycle.

Both HydA1 C169S and HydA1^odt^ have been shown to be largely inactive^4^,^15^,^23^,^25^,^26^,^28^,^32^, and a definite proof for the catalytic relevance of H_hyd_ can only be given by monitoring the succession of states during catalysis at sub-turnover time resolution as previously done for [NiFe]-hydrogenase^16^. However, this work strongly suggests that H_hyd_, as identified by Mössbauer and nuclear resonance vibrational spectroscopy, corresponds to the first transient state in the catalytic mechanism of H_2_ oxidation and is shared by all major subtypes of [FeFe]-hydrogenases. It is therefore plausible to assume that we successfully accumulated a state carrying a terminal hydride ligand in native [FeFe]-hydrogenases. In response to modulating substrate and product concentrations (H^+^/H_2_), accumulation of H_hyd_ was found to be fully reversible showing that H_hyd_ is no artificial dead-end state but part of the dynamic redox equilibrium typically occurring in the catalytically competent enzyme. All available data strongly suggest that H_hyd_ is indeed the missing link in the catalytic mechanism of [FeFe]-hydrogenases.

## Methods

### Site-directed mutagenesis of HydA1 and CpI

Plasmids pET21b-HydA1Cr and pET21b-CpI were used as templates for site-directed mutagenesis, which was performed according to the QuikChange-PCR protocol published by Zheng *et al*.^33^ For generating the constructs encoding HydA1 variants C169A, C169S and E141A, the 5′-overlapping mismatch primer pairs 5′-CAGCGCGTGTCCGGGCTGGATTGC-3′/5′-CCGGACACGCGCTGGTAAACATCGG-3′, 5′-CAGCTCATGTCCGGGCTGGATTGC-3′/5′-CCGGACATGAGCTGGTAAACATC-GG-3′ and 5′-CATCATGGAAGCGGGCAGCGAACTGCTGCATCGTC-3′/5′-GCAGTTC-GCTGCCCGCTTCCATGATGGTCAGATCC-3′ were used, respectively.

For substitutions C299A and E279A within the polypeptide of CpI, primer pairs 5′-CCTCTGCGTGCCCAGGTTGGGTACGTC-3′/5′-CCTGGGCACGCAGAGGTAAACATT-GGGAAAGGG-3′ and 5′-CCAGTTCGGTAGCCGCTTCCATAATGGTCATATC-3′/5′-TG-GAAGCGGCTACCGAACTGGTTCAACG were utilized.

### Heterologous expression of HydA1 and CpI proteins

The original plasmids pET21b-HydA1Cr and pET21b-CpI and their mutagenesis constructs were used for the heterologous expression of wild-type enzymes and variants of HydA1 and CpI (refs 4, 25, 34, 35). Apo-forms of CpI and HydA1 (lacking 2Fe_H_) were expressed anaerobically in *Escherichia coli* strain BL21 ΔiscR (ref. 36). Cells first grew aerobically in lysogeny broth (LB) medium pH 7.4 (0.1 M morpholineopropanesulfonic acid (Mops)-NaOH) supplemented with 5 g l^−1^ glucose and 2 mM ammonium iron citrate until an OD600 of 0.35–0.6 was reached. Before switching to anaerobic cultivation, the culture was flush with N_2_ for 30 min to remove residual O_2_ and supplemented with 25 mM sodium fumarate. Induction of gene expression was initiated by adding 5 mM cysteine and 0.5 mM β-D-1-thiogalactopyranoside. Cells were collected after 16–24 h expression via centrifugation.

### Purification of [FeFe]-hydrogenase apo-proteins

The apo-proteins of wild-type and mutant forms of HydA1 and CpI, containing the 4Fe_H_ subcluster but lacking the 2Fe_H_ moiety, were expressed under anaerobic conditions in *E. coli* strain BL21 ΔiscR as described earlier^4^,^34^,^36^. After cell disruption via ultrasonication and the separation of the soluble proteins from the cell debris by ultracentrifugation and subsequent filtering (pore size 0.2 μm), we exploited the C-terminally fused Strep-tag-II sequence (WSHPQFEK) for protein purification, performing affinity chromatography with Strep-Tactin Superflow resin (IBA GmbH) in 100 mM Tris-HCl buffer (pH 8) supplemented with 2 mM NaDT. Protein purity was verified by SDS–PAGE and protein concentration determined via Bradford assay (BioRad).

### Synthesis of 2Fe_H_ cofactor complexes

The native-like 2Fe_H_-cofactor-mimic complex [Fe_2_[μ-(SCH_2_)_2_NH](CN)_2_(CO)_4_][Et_4_N]_2_ (adt-2Fe_H_) and its odt derivative [Fe_2_[μ-(SCH_2_)_2_O](CN)_2_(CO)_4_][Et_4_N]_2_ (odt-2Fe_H_) were synthesized according to literature procedures^25^.

### *In vitro* maturation

To obtain the fully equipped holoprotein, heterologously expressed wild-type and mutant apo-proteins of HydA1 and CpI were maturated *in vitro* by adding adt-2Fe_H_ (odt-2Fe_H_) to a 10-fold excess in 0.1 M K_2_HPO_4_/KH_2_PO_4_ buffer (pH 6.8), supplemented with 2 mM NaDT. To ensure complete sample maturation, the reaction mix was incubated for 1 h at room temperature. The resulting holoproteins were cleaned from surplus 2Fe_H_ complex by size-exclusion chromatography using NAP 5 columns of GE Healthcare and concentrated in Amicon Ultra centrifugal filters 30K (Millipore). Afterwards, methyl viologen-specific H_2_ production activity was determined to assess catalytic competence.

### *In vitro* assay to determine H_2_ production activity

H_2_ production activities have been determined *in vitro* for wild-type and mutant proteins using 0.2 μg ml^−1^ of *in vitro* maturated [FeFe]-hydrogenase in 2 ml of 100 mM postassium phosphate buffer, pH 6.8, supplemented with 100 mM NaDT and 10 mM methyl viologen. Suba-seal vessels were sealed with stoppers, degassed for 5 min with 100% Argon and incubated for 20 min in a shaking water bath adjusted to 37 °C. For product quantification, 400 μl of sample head-space was analysed via gas chromatography (Shimadzu GC 2010). When enzyme activity was below the detection limit, the assay was repeated with a 10-fold increased enzyme concentration (2 μg ml^−1^).

### Crystallization and structure analysis of CpI variant E279A

Using a 1:1 mix of E279A holoprotein (10 mg ml^−1^) and reservoir solution (0.1 M MES, pH 6, supplemented with 0.4 M MgCl_2_, 24% polyethylene glycol and 16% glycerol) in a hanging drop vapour diffusion set-up, single cuboid-shaped crystals of brownish colour were identified after 2–4 days at 277 K under anaerobic conditions. The selected protein crystal was mounted into a CryoLoop (Hampton Research) and flash-frozen in liquid N_2_. Diffraction data were collected at 100 K at beamline ID23-2 of the ESRF in Grenoble, France and processed using the software package XDS^37^. Molecular replacement and structure optimization were achieved with the software packages PHENIX^38^ and Coot^39^. For details on crystallographic data of CpI E279A, see Supplementary Table 2.

### Infrared spectroscopy

All FTIR spectroscopy was conducted on a rapid-scan Tensor 27 spectrometer (Bruker Optik, Germany) equipped with a three-reflection ZnSe/silicon crystal ATR cell (Smith Detection, USA). The spectrometer was situated in an anaerobic gas chamber (Coy Laboratories, USA) in a water-free atmosphere of typically 99% N_2_ and 1% H_2_. Supplementary Fig. 1 depicts the process flow diagram of the experimental set-up. Approximately 5 bar nitrogen carrier gas of ultra-high purity (5.0) was provided by a PN1450 nitrogen generator (Inmatec, Germany). The exact amount of N_2_ was adjusted with a digital SmartTrak mass flow controller (MFC, Sierra, USA). Molecular H_2_ (H_2_ 5.0, Linde, Germany) was injected separately via a second flow controller to create a well-defined mixture. The gas passed a 200 mbar check valve to protect the flow controllers from humidity. Afterwards, two sequential wash bottles (H_2_O and miscellaneous content, for example, buffer) could be switched into the gas stream. A bypass loop allowed for an adjustable proportion of the gas to bypass the wash bottles entirely or to run through the liquid volume to create a mix of carrier gas, water vapour and microscopic drops of liquid water (aerosol). The aerosol stream was fed to a customized polychlorotrifluoroethylene (PCTFE) gas cell, screwed gas-tight onto the ATR crystal plate. An exit line eventually guided the aerosol to a gas dump (fume). The PCTFE gas cell was equipped with three inlets and a manometer (not shown in Supplementary Fig. 1). Directly over the silicon crystal of the ATR unit, a cylindrical cavity joined all inlets. A gas-tight lid was screwed on top of the gas cell and allowed irradiation via an acrylic glass window. At this position, an infrared- or ultraviolet/visible-transparent window with protein sample could be adjusted into the aerosol stream. Thus, transmission samples for, for example, ultraviolet/visible or Raman spectroscopy could be prepared under the same conditions as monitored via the ATR silicon crystal. All spectra were recorded with 80 kHz scanning velocity, at a spectral resolution of 1 cm^−1^, and varying extent of co-additions. All pH titrations were performed with citrate buffer (SSC).

### Data availability

The atomic coordinates and factors for the reported crystal structure for CpI variant E279A have been deposited with the Protein Data Bank (PDB) under accession code 5LA3. Further data supporting the findings of this study are available within the article and its Supplementary Information file and from the corresponding authors on reasonable request.

## Additional information

**How to cite this article:** Winkler, M. *et al*. Accumulating the hydride state in the catalytic cycle of [FeFe]-hydrogenases. *Nat. Commun.* **8,** 16115 doi: 10.1038/ncomms16115 (2017).

**Publisher’s note**: Springer Nature remains neutral with regard to jurisdictional claims in published maps and institutional affiliations.

## Supplementary Material

Supplementary Information
