# Supplementary material for: Accumulating the hydride state in the catalytic cycle of [FeFe]-hydrogenases
Source: Nat Commun. 2017 Jul 19;8:16115. doi: 10.1038/ncomms16115 (PMC5524980; doi:10.1038/ncomms16115)
Supplement: Supplementary Information [file ncomms16115-s1.pdf]

SUPPLEMENTARY TABLES

Supplementary Table 1| Survey of the determined state specific CN^-/CO-vibrational band patterns of HydA1, CpI and DdH wild type and mutant proteins.

State	*p*CN	*d*CN	*p*CO	*d*CO	*μ*CO
**HydA1**					
**Hhyd**	**2082**	**2068**	**1978**	**1960**	**1860**
Hox-blue	2092	2075	1970	1946	1812
Hox	2088	2072	1964	1940	1802
Hred	2072	2033	1915	1891	1961*
Hsred	2066	2026	1918	1882	1953*
**C169A**					
**Hhyd**	**2082**	**2068**	**1978**	**1962**	**1862**
Hox	2088	2072	1970	1938	1804
**HydA1odt**					
**Hhyd**	**2081**	**2076**	**1980**	**1962**	**1868**
Hox	2086	2070	1972	1948	1812
**DdH**					
**Hhyd**	**2089**	**2079**	**1980**	**1963**	**1860**
Hox-blue	2097	2082	1970	1946	1809
Hox	2093	2078	1965	1940	1802
Hred	2079	2040	1915	1892	1962*
Htrans[1]	2100	2075	1983	1977	1836
**CPI**					
**Hhyd**	**2082**	**2068**	**1984**	**1968**	**1856**
Hox-blue	2084	2073	1975	1953	1809
Hox	2082	2071	1970	1947	1800
Hred	2071	2053	1915	1899	1962*
Hsred	2065	2039	1922	1894	1958*
**E279A**					
**Hhyd**	**2082**	**2068**	**1984**	**1970**	**1858**
Hox-blue	2084	2073	1975	1952	1808
Hox	2082	2071	1970	1946	1800

* terminal CO in reduced states

Supplementary Table 2| Crystallographic data for CpI E279A

**Data collection**	**CpI-E279A**
Space group	P 1 2_1 1
Cell dimensions	
a, b, c (Å)	90.48, 73.58, 103.81
α, β, γ (°)	90.00, 96.29, 90.00
Resolution (Å)	48.46-2.29 (2.35-2.29)*
R_{merge}	0.1748 (0.8058)
I / σ(I)	8.18 (2.06)
Completeness (%)	99.1 (98.8)
Redundancy	4.9 (5.0)

**Refinement**	**CpI-E279A**
Resolution (Å)	48.46-2.29
No. reflections	60,779
R_{work} / R_{free}	0.1663 / 0.2127
No. atoms	
Protein	8918
Ligand / ion	106 / 2
Water	789
B-factors	
Protein	33.30
Ligand	19.40
Water	40.20
R.m.s deviations	
Bond lengths (Å)	0.004
Bond angles (°)	0.715

SUPPLEMENTARY FIGURES

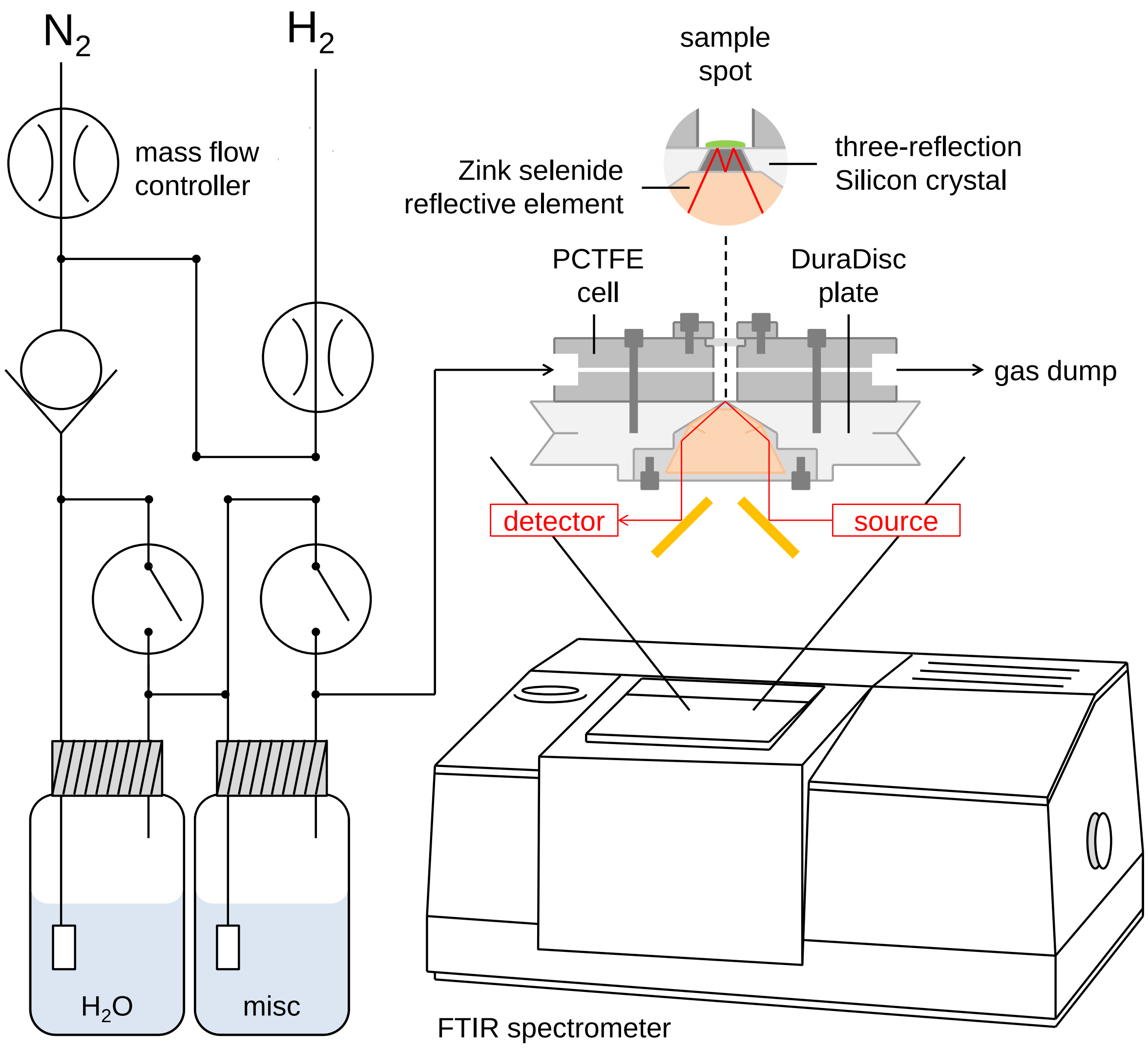

Supplementary Figure 1| Process flow diagram of the experimental set-up for ATR FTIR spectro-chemical titrations via N_2/ H_2 aerosol.

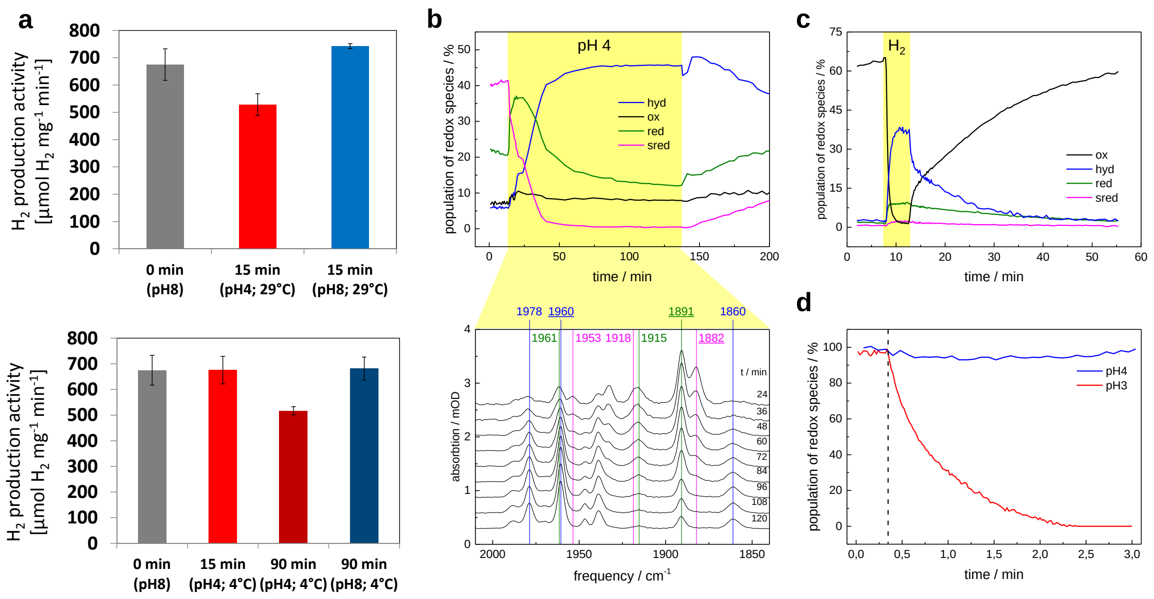

Supplementary Figure 2| Stability of HydA1 under pH4 and reversibility of pH and H_2 dependent changes in state fractions of HydA1 during ATR-FTIR titration experiments. **(a)** To assess the influence of buffer pH on enzyme stability, 400ng HydA1 was incubated in 100mM Tris-HCl of either pH8 (dark blue) or pH4 (red) for 15 min at 29°C (upper diagram) or up to 90 min at 4°C (lower diagram). Afterwards the sample pH was readjusted to pH6.8 by adding a surplus of 100mM potassium phosphate buffer (pH6.8) and the *in vitro* H_2 production activity of HydA1 was determined compared to untreated sample (grey) as described above. After exposing HydA1 in pH4 for 15min to elevated room temperature or 90min to 4°C, still more than 75% of the control activity can be determined. As HydA1 samples are highly concentrated (1-3mM) and kept at 4°C the slight inactivation effect determined here is of no significant consequence for the pH dependent and clearly reversible titration to H_{hyd} within the experimental setup of the above described ATR-FTIR-system. Error bars represent standard deviation of three independent measurements. **(b)** ATR FTIR experiment showing the population of redox species as a function of aerosol-mediated pH-titrations. Upper panel: curves track the relative intensity of prominent IR marker bands for H_{red} (1891), H_{sred} (1882), H_{hyd} (1960) and oxidized species. Lower panel: FTIR spectra from 25 to 120 minutes in the CO region of HydA1 detected under pH 4 (yellow mark-up). The species as plotted in graph B are assigned. **(c)** ATR-FTIR experiment demonstrating the reversible population of redox species of HydA1 at pH 4 and 2 mM dithionite as a function of N_2 or H_2 exposition (white and yellow mark-up, respectively). **(d)** Decrease of signal-intensity of the $2Fe_H$ cofactor ligand spectrum as function of aerosol-mediated pH-titrations. The curve tracks the relative intensity of oxidized species. The experiment was performed at ambient temperature (24°C climate control). In presence of N_2 and 500 mM dithionite at pH8 the sample is nearly 100% oxidized (1st phase). When the pH is shifted from 8 to 3 (2nd phase), fast and irreversible loss of cofactor signals can be observed. When instead adjusting the sample from pH8 to pH4 cofactor signals are retained, demonstrating sample stability under the chosen experimental conditions in contrast to measurements executed at pH3.

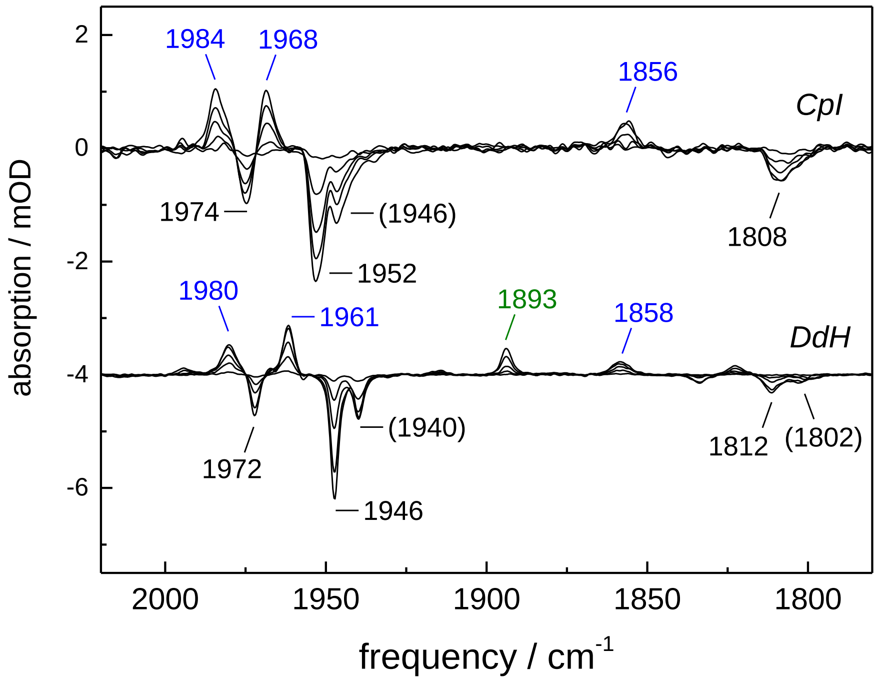

Supplementary Figure 3| Population of the transient H_{hyd} state in CpI and DdH under increasing H_2 pressure at pH4 showing that the H_{hyd} is a common feature of all [FeFe]-hydrogenases. Note that in spectra recorded at pH 4 the main carbonyl bands of the oxidized state were shifted by 6 – 10 cm^{-1} to higher frequencies presumably being a consequence of a protonation of H_{ox} at high proton pressure. This as yet unreported behavior resembles the one described for C169S[1,3] (see Supplementary Note 1). For the complete state specific CN^-/CO-vibrational spectra of DdH and CpI observed during sample analysis see Supplementary Table 1.

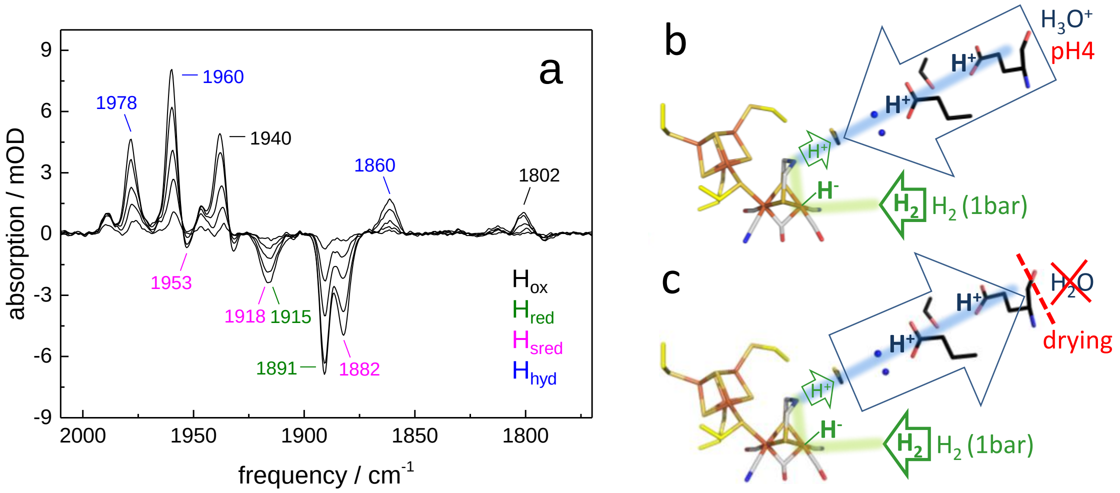

Supplementary Figure 4| Population of the H_{hyd} state at pH8 by combining H_2 gassing and dehydration. (a) ATR FTIR difference spectrum of HydA1 wild-type protein depicting the accumulation of H_{hyd} at the expense of the reduced states H_{sred}/ H_{red} under 1 bar of H_2 and decreasing sample humidity. **(b-c)** Both, high proton pressure (b, pH4) and lack of surface water **(c)** leads to an accumulation of protons in the proton transfer pathway which under high H_2 partial pressure results in a selective enrichment of the transient H_2 activated H_{hyd} state in wild-type [FeFe]-hydrogenase. For the complete state specific CN-/CO-vibrational spectra of HydA1 observed during sample analysis see Supplementary Table 1.

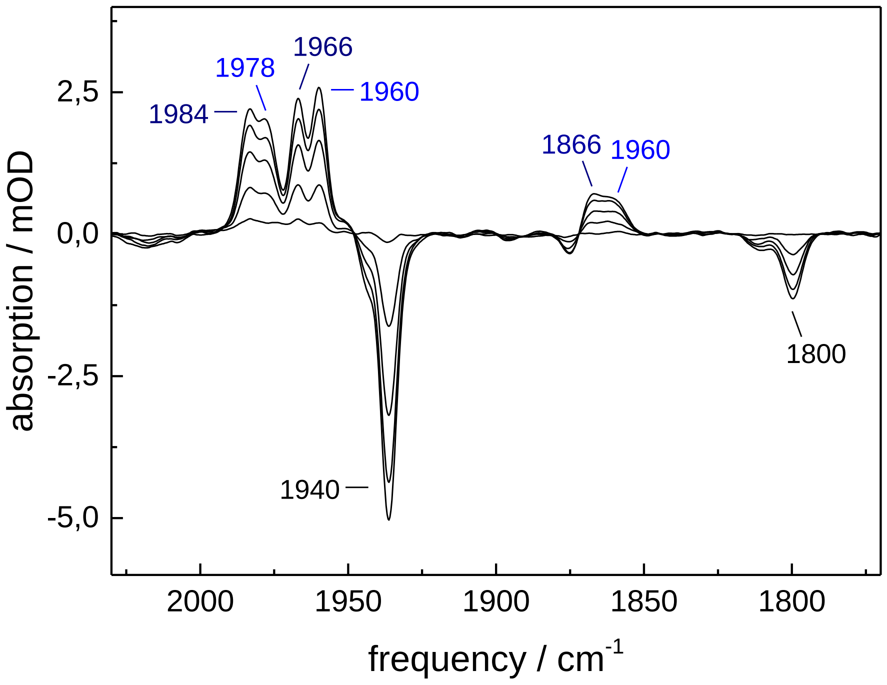

Supplementary Figure 5| HydA1 C169S shows two H_2 activated H_{hyd} states differing by a yet unknown protonation step. ATR FTIR difference spectrum of HydA1 variant C169S depicting the collinear enrichment of H_{hyd} (light blue) and a 'blue-shifted' H_{hyd} species (dark blue) at the expense of H_{ox} at pH 8 under H_2.

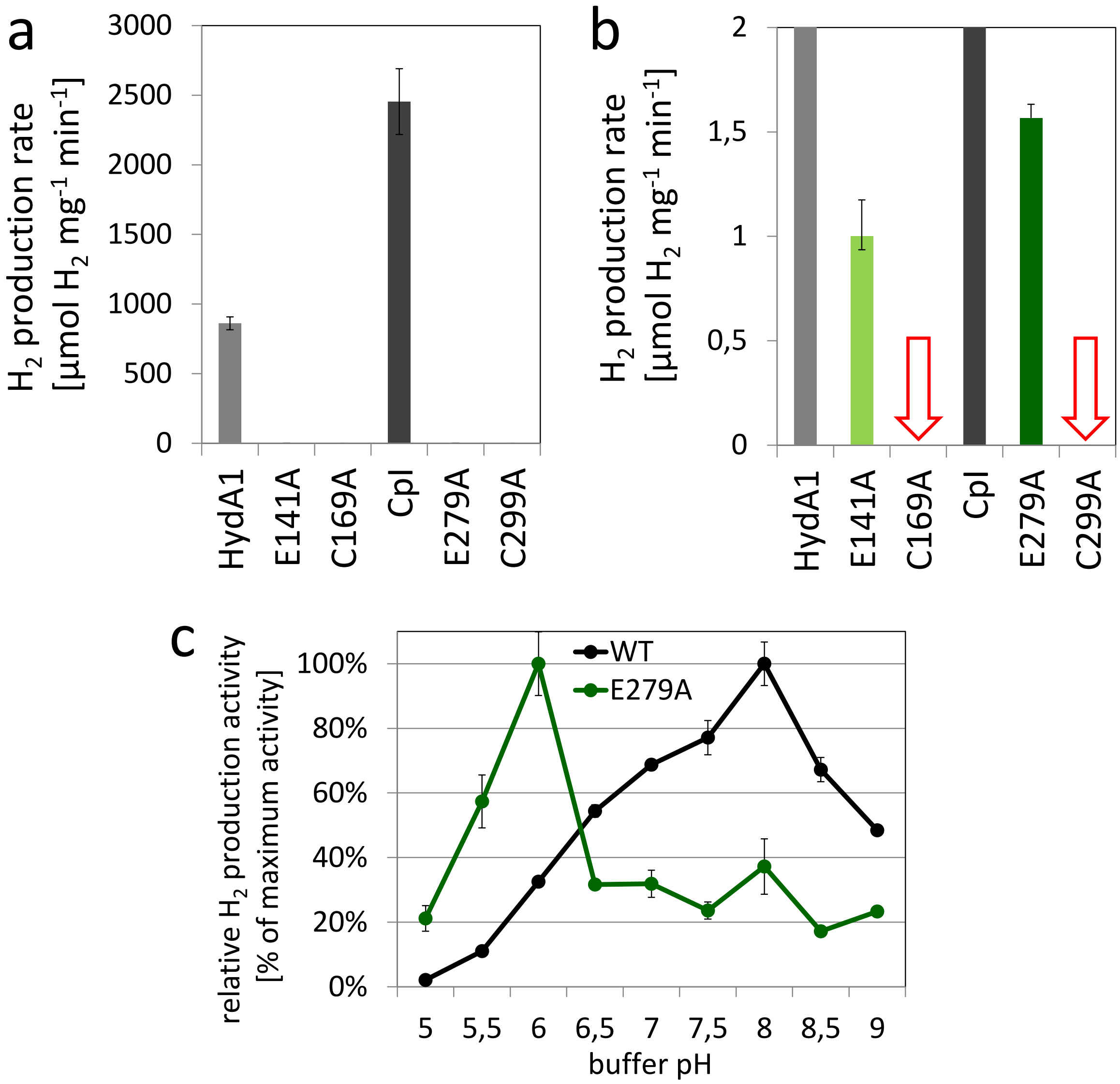

Supplementary Figure 6| H_2 production activities of site directed mutagenesis variants targeting the proton transfer pathway. (a) H_2-production activities of wild-type and single exchange variants of the PTP of [FeFe]-hydrogenases HydA1 and CpI. (**b)** Rescaled diagram to depict H_2 production rates below 2 µmol H_2 mg^{-1} min^{-1}. Red arrows indicate that the H_2-production rate is below the limit of detection. (**c)** pH-dependent enzyme activity of E279A and wild type CpI demonstrating a shift in the optimum pH by two units to the low pH range for variant E279A. This significant shift in pH-dependent behavior suggests that the loss of enzyme activity which accompanies the E to A substitution is partially rescued by an increased proton pressure, advocating for E279 to be involved in proton transfer. Error bars represent standard deviation of 3-4 independent experiments

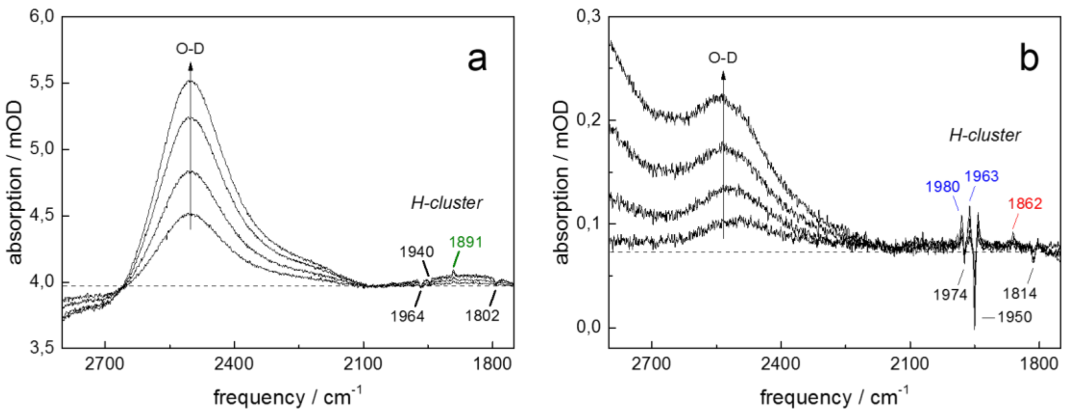

Supplementary Figure 7| D_2 uptake activity and HDO release of HydA1 and HydA1^{odt}. (**a**) Spectra show the immediate reaction of oxidized HydA1 wild-type to 0.1 bar D_2 at pH8 . Each spectrum comprises 15 sec of interferometer scans. Oxidation of D_2 induced a release of deuterium cations into the H_2O bulk solution and accordingly the O-D stretching bands at 2505 cm^{-1} arose. In the cofactor region, a decrease of H_{ox} marker bands (black) in favor of H_{red} (green) was observed. These changes are not well pronounced due to the minimal incubation time (here 60 sec). (**b**) Spectra show the reaction of an oxidized HydA1^{odt} sample film to 0.1 bar D_2 at pH 8. Each spectrum comprises 15 sec of interferometer scans. Oxidation of D_2 induced an increase of the HDO stretching band. In the cofactor region, a decrease of H_{ox} marker bands (black) in favor of H_{hyd} (blue) was observed. The µCO band of H_{hyd} (red) was shifted by 6 cm^{-1} to lower energies in comparison to the H_{hyd} pattern observed in as-isolated HydA1^{odt} (compare Fig. 3 and Supplementary Fig. 7). As an approximation the HDO release activity of wild-type HYDA1 is at least four orders of magnitude higher than HydA1^{odt}.

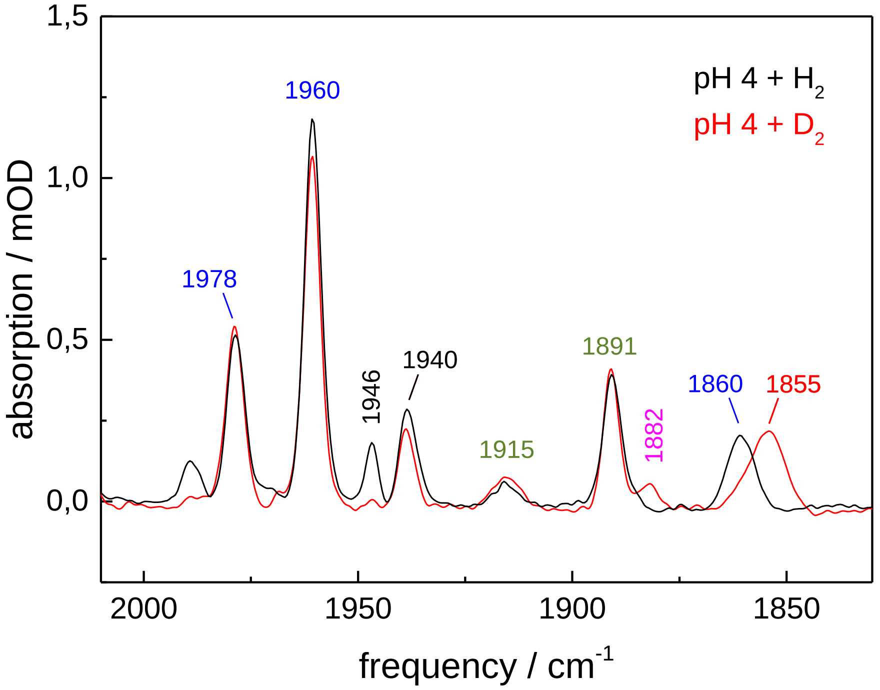

Supplementary Figure 8| H/D exchange supports the presence of a hydrido species in H_{hyd} of HydA1 wild-type. FTIR spectra from protein sample with H_{hyd} state (blue labels) accumulated at pH 4 and in presence of H_2 (black traces) or D_2 (red traces). Note the 5 cm^{-1} shift to lower frequencies of the µCO vibration in presence of D_2 (red label). Other redox species include H_{ox} (black), H_{red} (green), and H_{sred} (magenta).

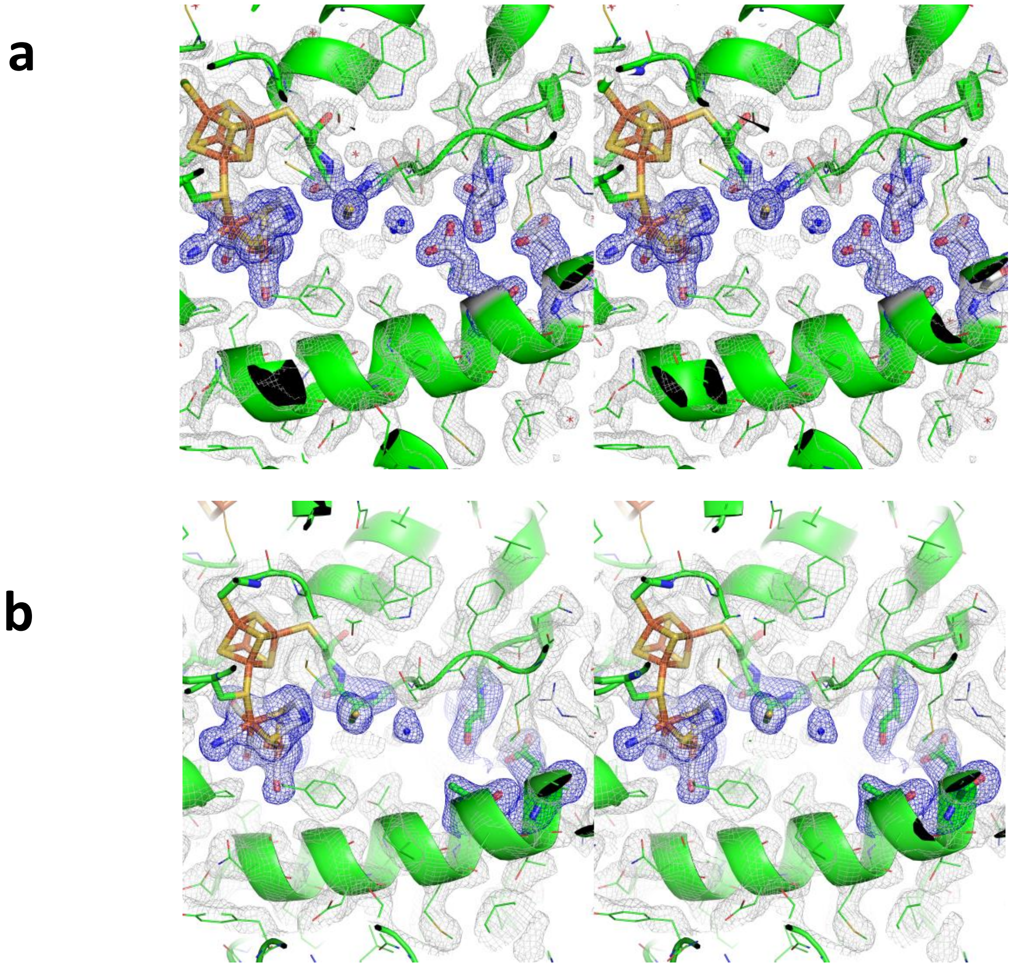

Figure S 9| Stereo view of the PTP in the crystal structures of wild type CPI and E279A. Electron density maps (blue) of the $2Fe_H$ cluster and the amino acid residues (stick structures) and H_2O-molecules (blue nb-spheres) involved in proton transfer, embedded in the 2Fo-Fc electron-density maps (grey) of the X-ray structures of CpI wild type **(a)** and variant E279A **(b)**. Stick models and simulated annealing omit |Fo-FcCrystal structure data for wild-type CpI correspond to pdb-database entry 4XDC. Details of the crystallographic data of E279A are summarized in Supplementary Table 2.

SUPPLEMENTARY NOTE 1

Previously we described a band pattern strongly resembling the one of the "super-oxidized" H_{hyd} state to be part of the FTIR spectrum of reduced sample of HYDA1 variant C169S[1] which consisted of two very similar states. EPR and FTIR spectroscopy allowed the identification of a redox state with a reduced $4Fe_H$ site (+1) and an over-oxidized $2Fe_H$ cluster (+4) resembling H_{trans} of DdH. However, our present data gained with DdH at pH4 and 1bar H_2 clearly show that H_{hyd} (1980/ 1963/ 1856 cm^{-1}) and H_{trans} (1983/ 1977/ 1836cm^{-1})[2] are two distinct states.

Mulder and coworkers reproduced and complemented the FTIR and EPR measurements for C169S and provided DFT data to support the presence of a hydride species further showing that the two states only deviate by their protonation state.[2] Supplementary Figure 4 demonstrates how the two states (1: 1860/ 1960/1978 cm^{-1} and 2: 1866/1966/1984 cm^{-1}) are accumulated under H_2 at pH8 in similar proportion at the expense of the former H_{ox} state. To probe the pH responsiveness of the equilibrium between states 1 and 2 we titrated C169S from pH8 to pH4 observing an increasing population of blue-shifted state 2 at the expense of state 1. This behavior recalls the pH-dependency of the blue-shifted H_{ox} state reported above and might be subject to the same mechanistic principle which is currently investigated by our laboratories. As described earlier for variant C169S, H/D exchange is expected to cause a selective shift in the vibrational band assigned to µ-CO provided the presence of a terminal hydride species positioned in trans to µCO.[3]

H/D exchange upon D_2 exposition can be monitored by following HDO accumulation as exemplified for HydA1 in Supplementary Fig. 7A. Supplementary Figure 8 shows the base-line corrected spectra of wild type HydA1 under H_2 or D_2. Water was replaced by D_2O in 10 – 15 minutes and wild-type HYDA1 was titrated to lower pH with 1% D_2SO_4. H_{hyd} was populated under H_2 just before the atmosphere was exchanged to D_2. While bands around 1980 and 1960 cm^{-1} were not affected by H/D exchange, the band at 1860 cm^{-1} showed a shift

to lower energies by 5-6 cm^{-1}. This shift does however not occur when changing the solvent from H_2O to D_2O under H_2 which demonstrates that the shift-inducing deuteride species positioned in trans of µCO originates from D_2 rather than from the solvent.

References Supplementary Data

1 Knorzer, P. *et al.* Importance of the protein framework for catalytic activity of [FeFe]-hydrogenases. *The Journal of biological chemistry* **287**, 1489-1499 (2012).

2 Roseboom, W., De Lacey, A. L., Fernandez, V. M., Hatchikian, E. C. & Albracht, S. P. The active site of the [FeFe]-hydrogenase from Desulfovibrio desulfuricans. II. Redox properties, light sensitivity and CO-ligand exchange as observed by infrared spectroscopy. *J Biol Inorg Chem* **11**, 102-118 (2006).

3 Mulder, D. W. *et al.* Investigations on the role of proton-coupled electron transfer in hydrogen activation by [FeFe]-hydrogenase. *J. Am. Chem. Soc.* **136**, 15394-15402 (2014).
